# Food Stress in Adelaide: The Relationship between Low Income and the Affordability of Healthy Food

**DOI:** 10.1155/2013/968078

**Published:** 2013-01-29

**Authors:** Paul R. Ward, Fiona Verity, Patricia Carter, George Tsourtos, John Coveney, Kwan Chui Wong

**Affiliations:** ^1^Discipline of Public Health, Flinders University, Adelaide, SA 5001, Australia; ^2^School of Social and Policy Studies, Flinders University, Adelaide, SA 5001, Australia; ^3^Health Promotion Branch, Department of Health and Ageing, Adelaide, SA 5000, Australia

## Abstract

Healthy food is becoming increasingly expensive, and families on low incomes face a difficult financial struggle to afford healthy food. When food costs are considered, families on low incomes often face circumstances of poverty. Housing, utilities, health care, and transport are somewhat fixed in cost; however food is more flexible in cost and therefore is often compromised with less healthy, cheaper food, presenting an opportunity for families on low incomes to cut costs. Using a “Healthy Food Basket” methodology, this study costed a week's supply of healthy food for a range of family types. It found that low-income families would have to spend approximately 30% of household income on eating healthily, whereas high-income households needed to spend about 10%. The differential is explained by the cost of the food basket relative to household income (i.e., affordability). It is argued that families that spend more than 30% of household income on food could be experiencing “food stress.” Moreover the high cost of healthy foods leaves low-income households vulnerable to diet-related health problems because they often have to rely on cheaper foods which are high in fat, sugar, and salt.

## 1. Introduction

People in low paying jobs, particularly those who have only casual employment, are underemployed, or are on a government pension for retirement, sickness, or acting as a carer, find a range of financial stressors confronting them, the most significant for this paper being food insecurity. As in many other countries, Australian consumers have had to accommodate to increases in costs of basic food [1]. During the financial years 2007-2008 alone, overall food prices rose by 3.9%, while some basic food prices rose more sharply: cheese by 14.2%, milk by 12.1%, poultry by 11.0%, and bread by 6.8% [2]. Food cost plays a significant role in mediating food choice among low socioeconomic status (SES) groups [1, 3, 4], who often have to cut back on food spending to make room for other essentials such as housing and utilities [5–8], leading to decreased food security [9]. This paper is predicated on the suggestion that the effects of food insecurity on families on low incomes may help to explain the higher prevalence of overweight in low-income populations.

### 1.1. Policy Context in Australia

Food costs jumped into the political limelight prior to the Australian 2007 federal election, with voters demanding government action to reduce prices. To honour preelection promises, the newly elected Labor government initiated a national inquiry into grocery pricing soon after taking office. However, following the release of the Grocery Pricing Inquiry Report [10] and the consequent launch of the government web site to monitor prices [11], critics considered there would be minimal, if any, impact on reducing prices [12, 13]. This is partly because Australia is not immune to the global and economic factors and natural disasters like floods, attributing to rising costs of basic foods [14], and partly because the inquiry outcomes did nothing to actually address food costs, especially healthy food in low SES areas.

In Australia, the National Preventative Health Taskforce (NPHT) described obesity as one of three priority action areas for better health, beside tobacco and alcohol consumption. It emphasised that addressing social inequalities in differential access to healthy food is fundamental to obesity prevention [15]. In doing so, the NPHT identified food insecurity as an important concern for low-income Australians and many at-risk groups and acknowledged the ensuing negative health consequences of inadequate access to healthy food.

Food security, within developed countries such as Australia, can be defined as the “ability of individuals, households and communities to acquire appropriate and nutritious food on a regular and reliable basis, and using socially acceptable means” [16]. Food insecurity, then, describes limited or uncertain ability to acquire appropriate foods in socially acceptable ways. This is not merely a lack of food, but also when people fear running out of food or are forced to make significant changes to their usual eating patterns due to economic constraints [17]. The 1995 National Nutrition Survey (the most up-to-date data in Australia) estimates that 5.2% of the population of Australia are “food insecure” [18]. 

Data collected in South Australia estimates the food insecurity rate to be higher at approximately 7% [19]. However, this increases among at-risk groups who have lower incomes including unemployed (11.3%), rental households (15.8%) [18], those identifying as Aboriginal or Torres Strait Islander (23%) [20], and recently arrived refugees (71%) [21]. Single parents are also considered an at-risk group with reported levels of food insecurity as high as 23% [9, 17]. Diets of food insecure people are likely to lack variety and be of poor quality with lower levels of micronutrients [22–25]. Against this backdrop of rising food costs, associations between food insecurity and lower socioeconomic status, the main focus of this paper is whether people from low-income areas are less likely to consume “appropriate and nutritious food” because of poor access to healthy foods.

Adequate access to a healthy food supply is one of the major determinants of food security. In developed countries, food insecurity is associated with obesity [17, 26] and obesity-related disease [27, 28], mainly due to an increased consumption of foods high in fat and or sugar that are typically cheaper, more available, heavily marketed, and simpler to prepare than healthy alternatives [29, 30]. The health consequences of food insecurity go beyond obesity and include nutrient inadequacy [31], with lower self-reported health [27] and compromised child health [32].

### 1.2. Food Costs and Food Security in Low Socioeconomic Status Populations

There are a number of established factors differentially expose certain members of the population to periods of food insecurity and the associated consequences, including poverty [33], rising food prices in Australia [34], higher food prices, and greater density of unhealthy food options in socially disadvantaged areas [35, 36], other financial obligations [7], employment status [37], rurality [38], lower educational attainment [39], and lack of access to private transport [40]. A major issue identified in previous studies is that low socioeconomic status (SES) groups are, in many cases, not able to afford to purchase a wide enough range of healthy foods to maintain good health [19, 29, 41, 42]. Thus, food affordability, influenced by a range of factors, the most salient being employment status, level of education, cultural influences, and lifestyle behaviours, isolation (geographic, social, and cultural), as well as age, and disability, may affect a person's ability to access healthy food, thereby potentially compromising their nutritional status [19].

In the UK, a survey to assess the eating habits and health status of people on low incomes [43] found that they tended to consume a poorer quality diet comprising more energy dense foods such as processed meat, full fat milk, sugar, and soft drinks than those on higher incomes. They also consumed less wholemeal products and vegetables than the general population. Other studies have found that affordability of healthy foods could be a primary reason for people from low SES backgrounds not choosing healthy food [9, 19, 38, 41]. It has also been suggested that the cost of healthier foods varies substantially from place to place [44, 45], thus impacting on affordability.

Food costs are not the only consideration when deciding what foods to purchase. Consumers confront increasing amounts of information on food every day and, in response, simplify food choice through coping strategies such as avoiding and favouring foods; vigilance; actively seeking and using food safety information; moderation and variety; common sense based upon previous personal experience or the experiences of significant others; or lack of concern [46]. Scientific evidence is often rejected leading to behaviour that has the potential to damage health [47]. In practice, food choice is not only driven by health concerns but also by routine; personal food preference; ethics; food cost; convenience and access; and by previous experience [48].

In addition, the taste of food is a central driver of food choice and consumption. Taste has been identified as being a significant contributor to food choice [49], particularly for younger people who have less immediate concerns with health [50]. Cultural and gender differences have been noted in the relative importance placed upon taste and health. Participants from countries such as the USA [51] and UK [52] place greater importance upon health concerns and less upon the pleasure of eating than participants in countries such as France, Belgium, and Finland [51, 52]. Likewise, women generally place less concern upon the pleasure of eating than men also demonstrating greater concern with the healthiness of food [51, 52]. A national survey of 1109 people in Australia found that 88% of respondents considered the taste of food before its price, with females and people on higher incomes more likely to do so [53]. In addition, 52% of respondents said that they considered the price of food before its health and nutritional benefits, with males, younger people, and people with lower educational qualifications more likely to do so.

In order to study the affordability of healthy food, this study investigated the affordability of a Healthy Food Basket (HFB) in metropolitan Adelaide. A HFB is a tool commonly used to assess the cost and affordability of healthy food. The assessment was conducted in high and low household income areas of Adelaide to examine which area level effects on the cost of healthy foods.

## 2. Methods

While there is no national Australian HFB, several HFBs have been developed in different states: the biennial Queensland Healthy Food Access Basket (QHFAB) survey [54], the periodic Illawarra Healthy Food Basket (IHFB) survey in New South Wales [55, 56], the Northern Territory Market Basket Survey (MBS) [57], and the South Australian survey on food cost, quality, and variety for rural areas [58]. Common features of these HFBs are the use of one standard reference family to calculate affordability and nutrient requirements based on Recommended Dietary Intakes (RDI) [59]. This limitation and its relevance to generalizing findings to a wider population have been noted [38, 44].

This study used the cross-sectional Victorian Healthy Food Basket (VHFB) survey methodology [60]. The VHFB set of methods was chosen for two key reasons: first, it uses four distinct types of reference family, developed by and considering the 2003 Family Characteristics Survey [61] and the 2001 Census of Population and Housing [62]. Second, being developed in 2007, the VHFB uses the Nutrient Reference Values (NRVs) released in 2006 instead of Recommended Daily Intakes (RDIs) to assess nutritional adequacy [63].

### 2.1. Choice of Locations and Types of Food Stores

For the current study, two supermarkets from the highest and lowest household income Census Collection Districts (CDs) from each Local Government Area (LGA) in metropolitan Adelaide were surveyed. A total of 61 supermarkets from 17 LGAs out of a total of 18 Adelaide LGAs were included. The City of Adelaide LGA had only two supermarkets which matched the selection criteria. Two LGAs (Prospect and Walkerville) were combined as there were only three supermarkets in each of these LGAs. The order of supermarket surveying was chosen using a random number generator. Two supermarkets refused to take part in the survey, and in each case the next supermarket on our randomized list was surveyed. When the LGA did not have enough supermarkets in the highest and/or lowest tertile CDs, adjustments were made in selection with those in the middle tertile, but closest to the extreme tertiles, being surveyed.

The supermarkets in the study were limited to the three leading supermarket chains in South Australia, Woolworths, Coles, and Foodland, based on an earlier study which indicated that families prefer to do the bulk of their food shopping in large supermarkets than in smaller corner stores, service stations, and delicatessens [40].

Specialty shops (defined as butchers and greengrocers for the purposes of this study) were also surveyed if they were located within a maximum of ten minutes walking distance of the selected supermarkets. Our inclusion of speciality shops was due to our concern that people may not “solely” buy food from supermarkets, but may also purchase from local butchers and grocers. Twenty-three supermarkets had a greengrocer and a butcher within a ten-minute walking distance, while the remaining supermarkets had either a greengrocer or a butcher (but not both), or none, of these specialty shops. In total, 27 greengrocers and 34 butchers were surveyed. For items costed in each of the different shopping venues, refer to the appendix.

### 2.2. Conducting the Survey

Three researchers were trained in the use of the Victorian Healthy Food Basket (VHFB) methodology and documentation tools. All data were collected within a narrow window of time in May 2009 to minimise potential seasonal variation in the price of foods.

A pilot test was conducted involving six supermarkets (two for each data collector) to assess any issues with the data collection process. The data collectors, with the rest of the research team, compared their findings, and an inter-rater reliability test was conducted to measure consistency across the six supermarkets surveyed and to ensure the internal consistency of the survey itself after variations in data collection had been discussed.

Out of 46 items included in the VHFB, the pilot test revealed that 38 items were clearly understood by the researchers, and the prices showed consistency across the board. Two items needed to be checked with the author of the VHFB to confirm package sizes to be surveyed. Two items needed discussion and agreement on a calculation for sizes which did not exist but were on the VHFB survey. The inter-rater reliability test proved to be a useful process, validating the survey as well as providing a forum for the researchers to clarify issues of difference and move to agreed principles, thereby ensuring a consistent process for conducting the survey. 

After the pilot test and the necessary adjustments were made, the data collectors sought “on the spot” permission, via a letter of introduction, from the owner or manager of each specialty shop or supermarket approached, just prior to the conduct of each survey. The letter of introduction emphasized confidentiality and an assurance that no individual store would be identified. The data collection process required all food prices to be recorded on the VHFB data collection sheet.

### 2.3. Product Selection

The selection of products for the Healthy Food Basket (HFB) was based on the VHFB methodology [60, 64]. Products were recorded according to the cheapest brand price in specified sizes of the food items listed in the VHFB. When the specified size was not available, the next smallest size was chosen, and the cost was multiplied upwards to match the specified size. If the next smallest size was not available, then the next largest size was selected, and the cost was multiplied downwards to match the specified size. In order to provide the cheapest but realistic HFB, generic brands were not chosen. Where a brand name was specified, only that brand of product was assessed, and if it was not available, the closest alternative was chosen. Finally, the regular price of items was used instead of special prices to reflect the standard cost of the HFB.

### 2.4. Reference Families

This study examined four household types of reference families, including a “typical” family (two parents plus two dependents), a single-parent family (one parent plus two dependents), a single adult, and an elderly retired pensioner. The reference families were the same as those used in the VHFB [60].

Many Healthy Food Basket surveys only take a limited household range into account when comparing costs. For example, the Illawarra HFB looks exclusively at a family of six. This study has the flexibility to look at a range of family types, providing a more tailored view of what it actually costs for different family or household types. The variations in affordability according to household type and income has proved to be very revealing especially when examining the cost of a Healthy Food Basket for people on welfare benefits (single adult on a government pension).

### 2.5. Assessment of Affordability

The cost of the basket was calculated for each reference family and priced according to the guidelines in the VHFB survey [60]. Affordability of the HFB was defined as the cost of the HFB as a percentage of household income. Two kinds of incomes were used to measure affordability. The first was based on government welfare payments for unemployed families (Table 1), while the second was based on Equivalised Disposable Household Income (EDHI) for South Australia (SA) 2005/2006 [65], which was adjusted to current values using Wage Price Index [66] rises since 2005/2006 (Table 2). The study assumes that high-EDHI households shopped in supermarkets in high-income areas and low-EDHI households in supermarkets in low-income areas.

### 2.6. Data Analysis

The data were analyzed with SPSS v17.0 for Windows (SPSS Inc., Chicago, IL, USA). Cost and affordability of the HFB were calculated for each reference family. Mean (standard error of the mean:mean (SE)) costs were compared between supermarkets in high- and low-income areas using the *t*-test. Affordability was calculated as a mean (SE) for high and low EDHI, assuming that high-EDHI families shopped in high-income household income areas and vice versa. Affordability for welfare payment receiving families was calculated as cost of the HFB as a proportion of income mean (SE) for each family type. Significance was taken as *P* ≤ 0.05.

## 3. Results

### 3.1. Cost of the Healthy Food Basket (HFB)

The mean cost (SD) of the HFB items sourced exclusively from supermarkets showed no statistically significant difference between high- and low-SES areas. When sourced from both supermarkets and specialty shops (greengrocers and butchers), again the data shows no statistically significant difference between an HFB from high- and low-SES areas.

These findings demonstrate that geographic location of supermarkets and speciality shops across metropolitan Adelaide does not appear to impact on the cost of the HFB. Our findings indicate, instead, that family type and income have the most significant impact in terms of the affordability of the HFB and therefore on food security. In other words, access to and affordability of healthy food supplies in metropolitan Adelaide are not so much dependent on relationship between location of shop (whether they are in high- or low-SES areas) as on the location of people on a social strata in relation to income.

Further data revealed that, from supermarkets only, the “typical” family in high-income areas would need to spend on average 8.9% of income on the HFB, while families in low-income areas would need to spend 28.3% of income. Thus people on low incomes would need to spend at least three times as much in terms of proportion of income as the amount spent by people on high incomes. Similar proportions between low and high income were obtained for the other reference family types (e.g., single-parent family: 25.6% versus 8.0%; single adult 18.6% versus 5.9%). As well, the “typical” family would need to spend more, as a proportion of income, than the other family types (8.9% to 28.3% of income), with the single adult spending the least, as a proportion (5.9% to 18.6%). These figures were almost identical when the HFB was costed in supermarkets plus specialty shops.

For families receiving welfare benefits, the proportion of income that would need to be spent on the HFB ranged from 17% to 34% of income. The “typical” family on welfare benefits would need to spend a larger proportion of their income on the HFB (33.0%) than the other family types (single parent family 29.1% and single adult 28.6%). The “elderly retired pensioner” on benefits would spend the smallest proportion (17.4%). It appears that the more people within a household, the higher the proportion of the welfare payments that would need to be spent to afford the HFB, suggesting that the welfare payment does not accommodate the increased family size in relation to the cost of the HFB. There was no statistically significant difference between the percentage of income that would need to be spent on the HFB when obtained from supermarkets or from supermarkets and specialty shops.

Pearson's correlations were then conducted between variables relating to the affordability for the different family types at both supermarkets and combined supermarkets and specialty shops. Very high correlation coefficients were found (between 0.88 and 0.99, *P* < 0.0001). Essentially, the Pearson's correlation found no difference in the affordability between supermarkets and combined supermarkets and specialty shops.

**Figure 1:**
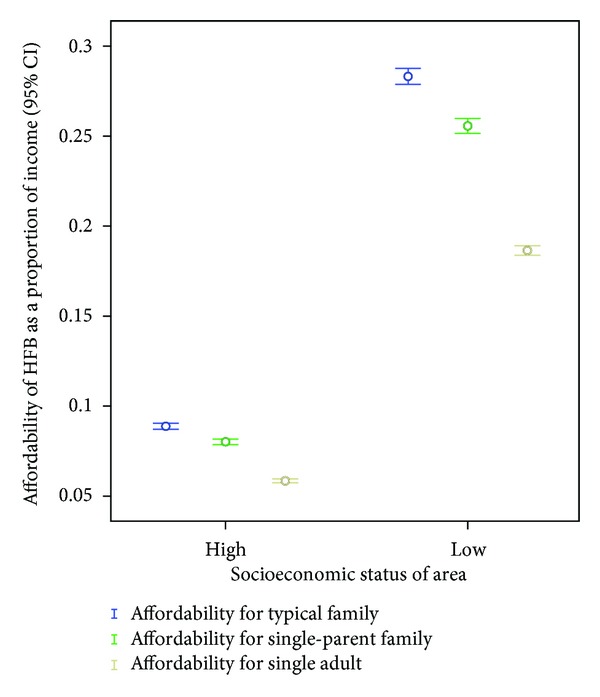
Affordability for different family types by SES.

Finally an error bar (Figure 1) was developed from the data which demonstrates that there is a clear distinction between values for family types in high-SES areas and the same family types in low-SES areas. More significantly, Figure 1 shows that the affordability of healthy food, as a proportion of income, differs according to family type. Regardless of socioeconomic status, the data points to healthy food becoming less affordable as the number of family members increases: healthy food is most affordable for a single adult, then a single parent with two dependents and is most expensive (least affordable as a proportion of income) for the typical family (two adults and two children).

## 4. Discussion

The findings showed that healthy food was significantly less affordable for families on low incomes where up to 28% of income would need to be spent to afford the HFB compared to high-income families (6% to 9%). On average, families in the lowest tertile earned approximately one third of the income of people in the highest tertile, explaining the difference in affordability between low- and high-income families.

For families on welfare payments, the situation was even worse. Excluding elderly pensioners, the percentage of income that would need to be spent on the HFB for the typical family, single-parent family, and single adult was 33.0%, 29.1%, and 28.6%, respectively. Similar results have been found in previous studies conducted in Adelaide [44], rural South Australia [38] and in the Illawarra region of New South Wales [67]. These findings suggest that in order to purchase a HFB, both low-income families and families on welfare payments would need to spend significantly more than the 17% average expenditure on food by Australian households. 

The findings also indicate that larger families have less income to be able to afford a healthy diet. Therefore, it appears that while a single person has to spread one income over a single person, a typical family (two parents plus two dependents) has to spread their two incomes over four people. This becomes another affordability factor, particularly for those “typical” families who may have only one income, or two low incomes. This “food stress” is a product of the cost of healthy food relative to the income of the household and is not due to the lack of access to healthy food. For low-income families and households this phenomenon cannot be separated from “housing stress,” occurring when households spend 30% or more of their income on housing costs [68–70]. The approximately 30% of income required to eat healthily (i.e., on the basis of the HFB) means that families on welfare payments may spend up to 60% of their welfare income on food and rent (or mortgage payments) before paying bills, transportation costs, educational costs for children, and medical expenses [71].

Such a scenario provides further understanding of the reasons why, in order to save money for other basic needs, families on low incomes tend to choose cheaper foods, which are often energy dense and nutrient poor [29, 72, 73]. Given the overall costs that families on low incomes face and given that housing is generally a fixed cost, low-income households are likely to attempt to save money on food, which is not a fixed cost, resulting in the purchase of unhealthy, energy dense, less expensive foods. This explains why people on low incomes inhabit an environment which may be classified as “obesogenic” for the simple reason that few healthy consumption options are open to them. It follows that educational messages alone aimed at the public about healthy eating will not change the unhealthy eating habits of people on low incomes who cannot afford to spend up to one third of their income to purchase a healthy diet. Reducing the price of healthy foods and raising welfare payments in the context of a national social policy framework could be the most direct and efficient ways to solve this issue [74]. Unaffordable healthy food on top of housing stress has serious consequences for health and wellbeing for low-income households.

### 4.1. Strengths and Limitations of the Study

One of the assumptions in this study was that people will shop at their local supermarket. There is some evidence from the USA that people on low incomes are willing to travel beyond their local food store in order to buy food, but this travel distance was between 1 to 1.5 miles [75]. However, research undertaken in Adelaide found that supermarkets were the main source of food shopping and that most people shop at one of their most local supermarkets [40, 76]. Nevertheless, whilst we assumed that people who live in a particular area are likely to also purchase food from their local supermarket, this may not always be the case.

It may also be the case that, in addition to the lack of affordability of healthy food for low income families, they may have to travel further to find supermarkets or other shops that stock healthy food, thereby adding a double-disadvantage associated with “food stress.” Our study did not examine the occurrence of the so-called “food deserts” in Adelaide, although they have been found in other Western countries [77–81]. Further research is needed in Australia to examine the nature and extent of food deserts, particularly in low-income areas. 

This study used HFBs as a “hypothetical” shopping list which, if purchased, would provide the necessary nutrition for the different reference families. In many studies using HFBs, methods are often not well described, and the rationale for and composition of the HFBs vary greatly. The constituents of the HFBs are meant to reflect the nutritional needs of population groups. However, some HFBs only use fruits and vegetables as a proxy for healthy foods whereas other HFBs are based on current food purchasing patterns obtained from household food surveys, which may not represent healthy food on the basis of nutritional guidelines [34, 82]. Some studies investigating the costs of HFBs have tried to be pragmatic by including healthier variations of popularly consumed foods [83–86] that do not attempt to encompass total dietary requirements. It has been argued that whilst these may be potentially more realistic in terms of what consumers may purchase, most appear to be quite subjective [83]. The HFB used on this paper was based on the Australian national nutritional guidelines and therefore represents the required nutrients to constitute a healthy diet. 

 In addition, HFBs may be criticized for being generic, and thus not necessarily encompassing healthy foods eaten as part of different culturally appropriate diets. For example, the range of vegetables eaten by different new migrant groups in Australia is not captured in the HFB, some of which may need to be purchased from speciality stores or home grown. We recognise this limitation of HFBs and suggest that further work needs to be undertaken to adapt HFBs to the culturally specific needs of particular new migrant groups. However, the HFB used in this paper was developed to represent the different dietary needs of different hypothetical reference families/households of various compositions and therefore attempts to understand the differential food affordability issues for such family types.

A key strength of our study is that we took into account data across the entire Adelaide metropolitan area (although two LGAs were collapsed into one due to small numbers of supermarkets in two of the LGAs). The previous HFB study in Adelaide [44] involved only five Local Government Areas (LGAs). However, this study has allowed us to examine patterns over the entire 18 LGAs of metropolitan Adelaide.

A further potential limitation of HFBs is that the prices of fresh food items in the HFB fluctuate during the year according to season and supply. While a one-time point measurement may not represent the average price of these food items, the HFB is a monitoring tool, and the fruit and vegetable items included in the HFB are generally available all year round. Given that food prices have been rising overall, there is value in establishing an ongoing monitoring system for South Australia to assist in assessing changes in the affordability of healthy food over time, as is the case currently in Queensland and the Northern Territory. Continuous measurement of the HFB would assist in developing an overall price index. Such an index could be used to compare changes in cost with income over time which would allow for the monitoring of the affordability of the HFB and would thereby lead to a greater understanding of the dynamic nature of food affordability over time.

## 5. Conclusion

Overall, affordability was a significant issue for families on low incomes in comparison to high-income families. The evidence come out of this study shows that the purchase of the HFB would create significant “food stresses” for families on welfare payments and low incomes. On top of housing stress that is already experienced by this population group, many of these people find themselves in extremely difficult economic circumstances. The food security and obesity literature, referred to earlier in this paper, points to the same associations, where lack of income is a barrier to purchasing healthy foods and correlated with overweight and obesity. The findings arising from this study solidify the link between income and cost resulting in “food stress” for people on low incomes. When linked to “housing stress,” it could be argued that people will scrimp on the more expensive healthy foods and spend more on the cheaper, often energy dense nutrient poor foods. These trends are also supported by previous work conducted by the authors of this paper in which a link was made between food insecurity and obesity [19]. Overall, this study has provided a valuable insight into the links between food cost and income and therefore food affordability.

## Figures and Tables

**Table 1:** Australian government welfare benefit payments^a^ (as of May 2009) per fortnight according to family type.

Typical family	Single-parent family	Elderly retired pensioner	Single adult
$1253.50	$975.88	$569.80	$453.30

^a^Australian government welfare benefit payments are paid to people who are either unemployed and looking for work, on disability payments, retired from employment, unemployed single-parent families, on sickness allowance, or on carer's allowance.

Note: data extracted from Department of Human Services—Centrelink site at: http://www.centrelink.gov.au/.

**Table 2:** Adjusted^a ^Equivalised Disposable Household Income for extreme tertiles per fortnight according to family type^b^.

	Typical family	Single parent family	Single adult
Lowest tertile	$1457.53	$1110.50	$694.06
Highest tertile	$4664.08	$3553.59	$2220.99

^a^Adjusted for Wage Price Index from 2005/2006 to March 2009.

^b^Data for elderly retired pensioner was not included as all elderly were assumed not to receive any income except welfare payments.

Note: data extracted from Australian Bureau of Statistics figures not in the public realm.

**Table 3:** Components of healthy food basket costed in various shopping venues.

Basket items	Product size	Costed in supermarkets	Costed in specialty shops (greengrocers)	Costed in specialty shops (butchers)
Cereal group				
White bread	650 g	X		
Wholemeal bread	650 g	X		
Crumpets (rounds)	300 g	X		
Weet-bix	750 g	X		
Instant oats	500 g	X		
Pasta	500 g	X		
White rice	1 kg	X		
Instant noodles	85 g	X		
Premium biscuits	250 g	X		
Fruit				
Apples	Per 1 kg	X	X	
Oranges	Per 1 kg	X	X	
Bananas	Per 1 kg	X	X	
Tinned fruit salad, natural juice	450 g	X		
Sultanas	375 g	X		
Orange juice (100%) NAS	2 L	X		
Vegetables, legumes				
Tomatoes	Per 1 kg	X	X	
Potatoes	Per 1 kg	X	X	
Pumpkin	Per 1 kg	X	X	
Cabbage	Half	X	X	
Lettuce	Whole	X	X	
Carrots	Per 1 kg	X	X	
Onions	Per 1 kg	X	X	
Frozen peas	Per 1 kg	X		
Tinned tomatoes	400 g	X		
Tinned beetroot	450 g	X		
Tinned corn kernels	440 g	X		
Tinned baked beans	420 g	X		
Meat and alternatives				
Fresh bacon, short cut, rindless	Per 1 kg	X		X
Fresh ham	Per 1 kg	X		X
Beef mince, regular	Per 1 kg	X		X
Lamb chops, forequarter	Per 1 kg	X		X
Chicken fillets, skin off	Per 1 kg	X		X
Sausages, thin beef	Per 1 kg	X		X
Large eggs (min 50 g, caged)	700 g dozen	X		X
Tinned tuna (unsat. oil)	425 g	X		
Tinned salmon, pink (water)	210 g	X		
Dairy				
Fresh full-cream milk	1 L	X		
Fresh reduced-fat milk	2 L	X		
Reduced-fat flavoured yoghurt	1 kg tub	X		
Full-fat long-life milk	1 L	X		
Cheese, block	500 g	X		
Noncore foods				
Polyunsaturated margarine	500 g	X		
White sugar	1 kg	X		
Canola oil	750 mL	X		

## Appendix

See Table 3.
